# P-238. Insights from Gut Microbiome Profiling: Evtepia spp. as a Distinctive Marker of Leanness in People with HIV

**DOI:** 10.1093/ofid/ofaf695.460

**Published:** 2026-01-11

**Authors:** Mackenzie K Joe, Isabella Adachi, Mariano J Lodigiani, Gilhen Rodriguez, Karen J Vigil

**Affiliations:** McGovern Medical School, Houston, Texas; McGovern Medical School at UT Houston, Houston, Texas; UTHealth, Houston, Texas; University of Texas McGovern Medical School, Houston, Texas; UT Health Science Center, Houston, TX

## Abstract

**Background:**

Obesity is a comorbidity in people with HIV (PWH) that complicates disease management and overall health outcomes. Emerging evidence suggests that the gut microbiome plays an essential role in metabolism, immunity, and inflammation, potentially influencing obesity status. Similarly, the specific microbial genera associated with obesity versus leanness among PWH remain insufficiently understood.

**Methods:**

Gut microbial diversity was assessed in 40 people living with HIV (PWH), classified into obese and lean groups based on body mass index (BMI). The microbiome was analyzed using next-generation sequencing (NGS) targeting the 16S rRNA gene. Differences in microbial genera abundance between groups were statistically evaluated using ANOVA with Tukey’s post-hoc analyses, complemented by alpha and beta diversity analyses performed with QIIME2.

**Results:**

While previous studies demonstrate a link between obesity and changes in gut microbial diversity, our study did not observe significant differences in alpha or beta diversity across BMI groups. However, we identified a unique association of *Evtepia spp.* with lean body status. In total, 27 genera exhibited significant differences based on their overall average mean scores. Of these, *Evtepia spp.* uniquely demonstrated a significant difference between obese and lean individuals, with a higher relative abundance in the lean group (Lean: 0.156, Obese: 0.028, Control: 0.000; p = 0.029). The remaining genera differed significantly across groups overall but were not specifically associated with obesity status.

**Conclusion:**

Through this study, *Evtepia spp*. emerged as a distinctive microbial genus significantly associated with lean body status in PWH, suggesting a potential role of this genus in metabolic health. The presence of beneficial gut microbiota like *Evtepia spp*. could help mitigate these metabolic disturbances by promoting better energy metabolism and reducing adiposity. Further research is needed to explore specific interventions that can enhance the presence of beneficial bacteria like *Evtepia spp.* in PWH.

**Disclosures:**

Karen J. Vigil, MD, Gilead: Advisor/Consultant|Gilead: Honoraria|Theratechnologies: Grant/Research Support|ViiV: Advisor/Consultant|ViiV: Grant/Research Support

